# One Health in Agricultural Sectors in Thailand, Lao PDR, and Vietnam: Interconnectedness Between Awareness and Socioeconomic Factors

**DOI:** 10.3389/ijph.2024.1607088

**Published:** 2024-11-28

**Authors:** Watchara Pechdin, Oulavanh Sinsamphanh, Long Bui-Thanh, Jiraphan Naruepatr, Sorasich Swangsilp, Mahesh Chougule, Ketkesone Phrasisombath, Tien D. N. Ho, Van-Phuc Phan

**Affiliations:** ^1^ Faculty of Social Administration, Thammasat University, Bangkok, Thailand; ^2^ Faculty of Environmental Science, National University of Laos, Vientiane, Lao People’s Democratic Republic; ^3^ Faculty of Economics, Tra Vinh University, Tra Vinh, Vietnam; ^4^ Department of International Relations, Faculty of Political Science, Chulalongkorn University, Bangkok, Thailand; ^5^ Faculty of Graduate Studies, University of Health Sciences, Vientiane, Lao People’s Democratic Republic; ^6^ Faculty of Economics and Law, Tian Giang University, My Tho, Vietnam; ^7^ School of Political Science, Can Tho University, Can Tho, Vietnam

**Keywords:** socioeconomic factors, one health, agriculture, farmer, awareness, ASEAN

## Abstract

**Objective:**

The study aims to analyze the interconnectedness of farmers’ socioeconomic factors and their awareness of the One Health framework.

**Method:**

This study conducted a survey with 1,166 observations across Thailand, Lao, and Vietnam and employed binary logit regression for data analysis. Odds ratios were used for interpreting the results.

**Results:**

The results indicated that certain socioeconomic factors—particularly household income, age, gender roles within the household, and household size—significantly influenced farmers’ awareness and engagement with One Health literacy. Awareness levels varied across the three countries: for example, the composition of adults in Thai farming households was correlated with awareness of environmental health and infectious diseases. In Lao PDR, gender was significantly associated with awareness of animal health, while in Vietnam, it was linked to awareness of animal disease transmission.

**Conclusion:**

These determinants contribute to the application of a more integrated One Health approach among farmers in these areas.

## Introduction

The One Health (OH) concept has emerged as a crucial strategy in addressing global public health challenges, particularly in the context of agricultural development. This approach emphasizes the interconnectedness of human, animal, and environmental health, recognizing that agricultural activities, especially those involving livestock, play a significant role in the transmission of infectious diseases [1–4]. Global recent research have attempted to advocate OH strategies for farmers to reduce this risk of disease transmission at the human-animal-environment interface, such as promoting good hygiene practices [5–7], monitoring wildlife health [8, 9], and ensuring sustainable agricultural practices [8, 10].

Awareness and socioeconomic factors were significantly found as crucial connection for the effectiveness of health interventions and advocacies [11]. Awareness ensures that communities understand the interconnectedness of human, animal, and environmental health, which is essential for achieving One Health goals. Socioeconomic conditions, on the other hand, affect access to resources and support systems necessary to implement these interventions effectively. In areas with limited resources, even well-designed programs may face challenges, while in communities with better socioeconomic standing, there is often more capacity to adopt and sustain One Health practices. Existing studies have found that individual awareness frequently covariates with socioeconomic and personal profile such as gender that woman may experience a more serious situation of infectious diseases than men [12–15], higher age [14, 16–18], large family composition [19–22], as well as lack of financial resources [21–23]. Nonetheless, with a shortage in-dept analysis, there is limited research on how socioeconomic factors potentially influencing awareness of OH in the southeast Asian context, in which numerous existing studies are more likely concentrated in the global North [7–12]. This puts the southeast Asian region at risk for future infection disease as effective mechanisms are not in order yet.

Particularly, farmers in Thailand, Lao PDR, and Vietnam find themselves vulnerable to those risk of infectious diseases, exacerbated by a combination of diverse socioeconomic factors. Limited financial resources, making them insufficient access to education and training, have left these agricultural communities ill-equipped to address and mitigate the threats posed by diseases originating from livestock or poor environment [24–26]. Moreover, economic fluctuations in these regions such as transforming agrarian landscapes, agricultural price fluctuation, or erratic weather have created additional challenges, potentially pushing farmers into cycles of poverty [11]. This condition underscores the urgent need for comprehensive support OH systems and interventions to enhance the resilience of these farmers in the face of potentially emerging infectious diseases.

This study aims to investigate the relationship between socioeconomic factors and farmers’ awareness of One Health principles, with a particular focus on Thailand, Lao PDR, and Vietnam. The goal is to provide a deeper understanding of how socioeconomic conditions such as gender, financial resources, age, family composition, and other demographic factors influence farmers’ awareness of One Health, which may help guide policymakers in developing more effective strategies for promoting One Health [4, 27, 28]. By comparing these countries, in addition, it could enhance current understanding on One Health approaches and the variation of its determinants in this region.

## Methods

### Study Populations and Data Collection Procedure

The study aims to investigate the factors influencing farmers’ awareness in three countries: Thailand, Lao PDR, and Vietnam. A structural questionnaire was used for data collection in those countries. Awareness of the farmers was measured by Likert scale through the questionnaire that we have developed and tested by Index of Item-Objective Congruence (IOC) by 10 health experts in Thailand (4), Lao (3), and Vietnam (3). Upon completion, the developed questionnaire was distributed to the three countries for pilot testing and validation, with 40 respondents participating from each country. The pilot results from each country can obtain Cronbach’s alpha greater than 0.70 that shows applicable to collect the data [29].

The study was conducted in Nakhon Phanom Province in Thailand, Savannakhet Province in Lao PDR, and the Mekong Delta (Can Tho, Tra Vinh, and Tien Giang) in Vietnam. These areas were selected because of their cultural, agricultural, and socioeconomic similarities, which enhance the reliability of cross-comparisons [14–16]. Within the constraints of the budget, a total of 1,166 individuals participated in face-to-face interviews using simple random sampling. These participants (n) comprised 300 respondents from Thailand (N = 559,000), 399 from Lao PDR (N = 1,117,500), and 467 (N = 4,008,854) from Vietnam. The sample size selection bias was less than 7% for all countries as determined by the Yamane sampling method [30];

$$n=\frac{N}{1+N\left(e^{2}\right)}$$

where n represents the sample size, N denotes the population as obtained from the recent census data provided by the City Population database [31], and e indicates the selection bias.

Participating farmers were required to be self-employed smallholders between the ages of 18 and 59. They were recruited on a voluntary basis who can provide sufficient information while conducting the interview and the consents for research activities. The proposal, data collection protocol, as well as questionnaire, were approved by the Human Research Ethics Committee of the authors’ affiliation.

According to the data collection process, the research team firstly contacted the local authorities and the village leaders to obtain permission and general information about study sites. Next, the village leader helped recruit the local volunteer for data collection and helped list potential respondents who were available on the day of making interview and satisfied with the requirements of the project.

The data was collected from October to November 2023. Prior to data collection, the volunteers underwent training covering various aspects, including not only topics related to One Health (OH) literacy, such as the intricacies of OH initiatives and their link to infection diseases, but also ethical practices to be observed during data collection from respondents. The training, lasting 4 hours, provided volunteers with an opportunity to address any doubts they had regarding the questionnaire. On the day of data collection, volunteers were mandated to elucidate the understanding of the One Health approach to respondents before proceeding. Data collection commenced only after ensuring that respondents had a clear understanding of the OH concept. To maintain data quality, research assistants were presented in the study area during data collection, assisting the volunteers in addressing complex questions.

### Measures

According to the developed questionnaire, five measurements of OH awareness were investigated into its socioeconomic predictors in order to unveil significant factors that could potentially enhance OH approach to farmers. These measurements include:

• O1: Basic OH concept (I believe that human health, animal health, and the environment are interconnectedness)
• O2: Collaborative work and health promotion (I believe that collaboration in different areas (e.g., livestock, agriculture, public health) are essential to the effectiveness of farmers’ health operations.)
• O3: Environmental health and infectious diseases (I believe that climate change has health implications, such as rising temperatures that could increase mosquito breeding and tick habitats.)
• O4: Animal health (I believe that diseases that affect animals can also affect the health of people.)
• O5: Animal disease transmission (I believe that solving animal health problems is essential to human health and wellbeing.)

### Independent Variables

Independent Variables for all measurements consisted of gender, age, household income, number of adults and number of children in the household.

### Analysis

Given the clustered study design, “Stata software version 18.0” was used for data analysis. Descriptive statistics (e.g., frequency, percentage) were employed to summarize socioeconomic characteristics of respondents and outcome variables. In order to detect multicollinearity, variance inflation factor (VIF) was adopted and the result reveals that no significance of multicollinearity was observed. Logistic analyses were utilized to examine the relationships between perception of respondents on One Health literacy (dependent variables) and their predictors by considering Odd-Ratio (OR). P-values of 0.01, 0.05, and 0.10 were considered as a significant level.

## Results

### Respondents’ Socioeconomic Profile

Table 1 shows the socioeconomic characteristics of 1,166 respondents in Thailand (300), Lao PDR (399), and Vietnam (467). On average, household income about 150–300 USD/month were found commonly in Thailand (46%) and Lao PDR (49%), whereas that was higher of above 450 USD/month in Vietnam (66%). Gender equality was concerned in this study; hence, the number of female respondents was nearly half across the three countries. At least 53% of respondents were heads or co-heads of households. More than 50% of respondents in all countries were an older adult (50 years old and above). More than 50% of households had at least one child and more than two adults (See Table 1).

**TABLE 1:** Respondents’ socioeconomic characteristic (Thailand, Lao PDR, Vietnam, 2023).

Covariates	Respondents					
	Thailand (n = 300)		Lao PDR (n = 399)		Vietnam (n = 467)	
	N	%	N	%	N	%
Household income (USD/month)						
Below 150	86	28%	152	38%	38	9%
150–300	137	46%	196	49%	118	25%
Above 300	77	26%	51	13%	311	66%
Gender						
Female	179	59%	184	46%	215	46%
Male	121	41%	215	54%	252	54%
Male being as head of household						
No	140	47%	122	31%	172	37%
Yes	160	53%	277	69%	295	63%
Age (years old)						
Below 40	43	15%	91	23%	154	33%
40–49	101	33%	104	26%	100	21%
≥50	156	52%	204	51%	213	46%
Total number of. children under 18 years Old in household (people)						
No Children	158	53%	171	43%	230	49%
1	97	24%	97	24%	169	36%
≥2	55	33%	131	33%	68	15%
Total number of adults above 18 years old in household (people)						
1-2	108	36%	126	32%	137	29%
3-4	173	58%	149	37%	284	61%
>4	19	6%	124	31%	46	10%

Source: Authors’ estimation using survey data (2023).

### Determinants of One Health Awareness

Determinants of One Health literacy among farmers and its significant correlation with socioeconomic factors were observed in each country. In Thailand, the understanding of the One Health (OH) concept (O1) was highly positively correlated with farmers who had household income 150–300 USD/month (OR = 1.82), were aged 50 years old or older (OR = 2.32), and had families with more than one child (OR = 4.08), when comparing to its based categories. In addition, partial respondents in Lao PDR with a monthly income above 300 USD/month and those with 3-4 adults in their family (OR = 0.34) significantly showed lower odds compared to their respective base categories (OR = 0.21 and 0.34, respectively). In Lao PDR, older age was found to be a significant factor, with individuals aged 50 years or older having significantly lower odds compared to younger individuals (OR = 0.35).

In addition, perceptions of positive collaboration (O2) were found to have a significant correlation with older age in the case of Thailand as well as monthly income and family size in Lao PDR. In contrast, all socioeconomic factors exhibited a weak relationship in Vietnam. However, several significant socioeconomic factors were identified in relation to environmental health (O3), animal health (O4), and animal diseases (O5). For instance, the composition of adults in Thai farmer’s family was correlated with O3, while age factors were significantly associated with animal diseases (O5). Further significant results were such as gender found significant with O4 in Lao PDR and O5 in Vietnam. Other details were indicated in Table 2.

**TABLE 2:** Determinants of One Health literacy among farmers (Thailand, Lao PDR, Vietnam, 2023).

Attitudes/Perceptions	Predictors	Thailand		Lao PDR		Vietnam	
		OR	*p-value*	OR	*p-value*	OR	*p-value*
OH concept (O1)	monthly income 150–300 USD	1.82*	0.10	0.59*	0.10	1.48	0.30
	monthly income 300 USD and above	1.64	0.30	0.21***	0.00	1.50	0.34
	gender (male)	1.34	0.41	0.67	0.27	0.85	0.53
	male being as head of household (yes)	1.32	0.43	0.82	0.65	0.92	0.78
	age 40–49 years old	2.32	0.11	0.89	0.78	0.42**	0.01
	50 years old or older	2.27*	0.10	0.86	0.74	0.35***	0.00
	have 1 child	0.91	0.80	0.96	0.91	0.88	0.59
	have more than 1 child	4.08**	0.04	1.22	0.60	0.88	0.68
	have 3-4 adults	n/a		0.37**	0.02	0.60	0.21
	have more than 4 adults	n/a		0.86	0.67	1.00	1.00
Effective collaboration among stakeholders (O2)	monthly income 150–300 USD	0.55*	0.09	0.72	0.26	1.01	0.97
	monthly income 300 USD and above	0.55	0.15	0.29**	0.01	0.86	0.72
	gender (male)	1.70*	0.09	0.71	0.33	0.88	0.61
	male being as head of household (yes)	0.76	0.36	0.72	0.42	0.79	0.44
	age 40–49 years old	3.10**	0.01	0.89	0.75	0.84	0.60
	50 years old or older	2.84**	0.02	1.25	0.59	0.72	0.27
	have 1 child	1.06	0.86	0.91	0.79	0.92	0.72
	have more than 1 child	2.42*	0.08	0.69	0.27	0.92	0.80
	have 3-4 adults	0.63	0.52	0.37*	0.02	0.83	0.65
	have more than 4 adults	0.43	0.22	0.29***	0.00	1.26	0.53
Interconnectedness of environmental health and infectious diseases (O3)	monthly income 150–300 USD	0.91	0.76	0.79	0.40	1.27	0.52
	monthly income 300 USD and above	0.87	0.73	0.24***	0.00	1.11	0.80
	gender (male)	1.38	0.27	1.47	0.23	0.75	0.24
	male being as head of household (yes)	0.89	0.69	0.42**	0.02	1.06	0.84
	age 40–49 years old	1.77	0.20	1.17	0.69	0.95	0.87
	50 years old or older	1.61	0.26	1.81	0.15	0.97	0.90
	have 1 child	1.06	0.86	1.10	0.77	1.22	0.38
	have more than 1 child	2.08	0.12	1.22	0.56	1.34	0.35
	have 3-4 adults	0.13*	0.06	1.14	0.73	0.74	0.45
	have more than 4 adults	0.13**	0.05	1.10	0.75	0.77	0.47
Interconnectedness of animal health and infectious diseases (O4)	monthly income 150–300 USD	0.79	0.48	0.70	0.15	0.86	0.70
	monthly income 300 USD and above	0.52*	0.09	0.81	0.62	0.89	0.79
	gender (male)	0.98	0.94	0.55**	0.05	0.76	0.27
	male being as head of household (yes)	0.89	0.69	1.04	0.92	1.65*	0.07
	age 40–49 years old	3.18**	0.01	1.21	0.56	0.61*	0.10
	50 years old or older	2.30**	0.04	1.23	0.56	0.54**	0.03
	have 1 child	0.92	0.79	0.96	0.89	1.55*	0.06
	have more than 1 child	1.66	0.27	0.64	0.13	1.81*	0.07
	have 3-4 adults	0.29	0.13	0.95	0.88	0.49*	0.07
	have more than 4 adults	0.29	0.11	0.74	0.30	0.90	0.79
Interconnectedness of animal disease and human health (O5)	monthly income 150–300 USD	0.79	0.48	0.67	0.14	2.01**	0.06
	monthly income 300 USD and above	0.52*	0.09	0.45**	0.06	1.39	0.43
	gender (male)	0.98	0.94	1.24	0.47	0.51***	0.01
	male being as head of household (yes)	0.89	0.69	0.30***	0.00	1.44	0.24
	age 40–49 years old	3.18**	0.01	1.42	0.35	0.70	0.29
	50 years old or older	2.30**	0.04	1.24	0.57	0.53**	0.03
	have 1 child	0.92	0.79	0.66	0.18	1.10	0.70
	have more than 1 child	1.66	0.27	0.66	0.20	1.58	0.20
	have 3-4 adults	0.29	0.13	1.10	0.81	1.03	0.94
	have more than 4 adults	0.29*	0.10	0.78	0.39	1.02	0.96

Remark: based categories (=0) are household with: monthly income below 150 USD, female gender, male being as head of household (no), age below 40 years old, have no children, and have less than 3 adults. In addition, ***, **, and * represent significance at 1%, 5%, and 10% levels, correspondingly.

Source: Authors’ estimation using survey data (2023).

## Discussion

The correlation between OH literacy among farmers and various socioeconomic determinants has been investigated, revealing noteworthy associations with different awareness of OH concept in three different countries. These findings emphasize the needs for targeted interventions to enhance health literacy in diverse agricultural communities.

Positive correlation with higher monthly income levels of households in Thailand might be influenced by a potential economic dimension to OH literacy (O1), in which farmers with higher incomes may have better access to resources and healthcare, significantly encouraging health literacy [24–26]. Conversely, this effect may not hold true in Lao PDR, where individuals with lower incomes are more likely to be aware of the OH concept, possibly due to their heavy involvement in agricultural activities that encourage this recognition. In addition, correlation with specific age groups, particularly in farmers aged 40-49 in Vietnam, indicates that people’s understanding of one health concepts may change at different stages of their lives. One reason could be that farmers during this age are more adept at acquiring new knowledge, have greater access to up-to-date health information, and are generally more receptive to new ideas compared to their older individual [24–26].

The perception of positive collaboration (O2) among farmers is also identified as a key factor that can enhance the effectiveness of health operations within the agricultural community in Thailand and Lao PDR. The absence of a significant correlation between O2 and socioeconomic profiles in Vietnam might be explained by the fact that the positive collaboration is likely perceived as a universally beneficial factor in the context of one health operations [30, 32]. Regardless of the socioeconomic status of farmers, the recognition of the value of collaboration may be shared among farmers. In addition, farmers, irrespective of their socioeconomic backgrounds, may share common goals and objectives related to health operations [33, 34]. The understanding that collaboration is essential for addressing shared challenges and achieving collective health outcomes. It might be ingrained in farming community, creating a broad consensus that transcends socioeconomic differences [5–8]. However, with a unique context in Laos, the perception of OH as a collaborative approach shows a significant negative correlation with higher household incomes and larger family. Higher-income households may prioritize individual health and wellbeing over community health concerns, leading to a diminished perception of the importance of collaborative efforts inherent in the OH approach. Families with larger sizes might experience diverse health dynamics that lead them to focus on immediate family health needs rather than collective community health issues, reducing their engagement with collaborative One Health initiatives.

Among significant socioeconomic factors, the negative correlation between an higher income among Thai farmer family and interconnectedness to environmental health (O3) suggests that households with higher incomes are more unlikely to invest in and prioritize environmental health practices [21–23]. Considering several significant factors in Vietnam, the positive correlation between a gender of household head and the interconnectedness to animal health (O4), consistent with [35]. In southeast Asia countries, household leaders often have a substantial and direct influence on the decision-making process regarding financial decision and other important issues, including animal care. It potentially accelerates the engagement of monitoring practices for the health of livestock. Furthermore, this study found weak correlation between household structure and interconnectedness to animal diseases (O5) among the three countries. One reason might be explained by the fact that the awareness of animal health may stem more from shared community knowledge and resources rather than family composition. Households in these three countries may access similar information through community programs, local veterinary services, or media, regardless of their internal structure.

### Conclusion

This study aims to explore the determinants of One Health literacy among farmers and examine potential correlations between socioeconomic factors and the five key elements measuring One Health awareness in Thailand, Laos, and Vietnam. The results indicate that certain socioeconomic factors, particularly higher household incomes, gender roles within the household, and larger household sizes, play an influential role in shaping farmers’ awareness and actions related to their awareness of One Health literacy. These findings underscore the importance of considering the socioeconomic context in the design and implementation of health interventions for shaping the awareness and practices of farmer.

A significant limitation of this study is the lack of variation in geographical characteristics among the areas studied across the three countries, meaning observed correlations and determinants should be interpreted within specific regional contexts. Additionally, we excluded educational level from the analysis due to heterogeneity concerns. As we used simple random sampling to manage costs, most participated respondents had less diverse levels of education, resulting in a largely homogeneous sample that may impact result quality. Consequently, all findings in this study should be interpreted with caution.

To enhance the robustness of research and ensure a comprehensive understanding of one health adoption among farmers, future studies are recommended to conduct with larger and more geographically diverse samples. This could involve including participants from various regions with distinct socio-economic, cultural, and environmental characteristics. Moreover, scholars may explore variations in OH adoption among farmers engaged in different types of agriculture (e.g., crop farming and/or livestock rearing) to discern sector-specific determinants. This comparative approach can contribute to tailored interventions that address the unique needs of diverse agricultural practices.
